# Integrated transcriptomic and proteomic analyses reveal ɑ‐lipoic acid‐regulated cell proliferation *via* Grb2‐mediated signalling in hepatic cancer cells

**DOI:** 10.1111/jcmm.13447

**Published:** 2018-03-25

**Authors:** Lan Yang, Xiliang Wang, Juan Xu, Ya Wen, Manqiao Zhang, Jingxiao Lu, Rongfu Wang, Xiaojuan Sun

**Affiliations:** ^1^ Institute of Immunology of Zhongshan School of Medicine Sun Yat‐Sen University Guangzhou Guangdong China; ^2^ Shenzhen Tumor Immuno‐gene Therapy Clinical Application Engineering Lab Biobank of Shenzhen Second People's Hospital The First Affiliated Hospital of Shenzhen University Shenzhen China; ^3^ Department of Biochemistry of Zhongshan School of Medicine Sun Yat‐Sen University Guangzhou Guangdong China; ^4^ Shenzhen Xenotransplantation Medical Engineering Research and Development Center Institute of Translational Medicine Shenzhen Second People's Hospital First Affiliated Hospital of Shenzhen University Shenzhen Guangdong China; ^5^ Department of Pharmacology and Proteomics Center Zhongshan School of Medicine Sun Yat‐Sen University Guangzhou Guangdong China; ^6^ Department of Graduate School Guangzhou Medical University Guangzhou China

**Keywords:** ɑ‐lipoic acid, hepatocellular carcinomas, Grb2, RNA‐Seq, proteome

## Abstract

Hepatocellular carcinoma is the most frequent primary liver cancer worldwide. The use of antioxidants as cancer prevention and treatment agents has become a focus of research in recent years due to their limited adverse effects. Alpha lipoic acid (ɑ‐LA) is synthesized in the liver and is considered a naturally occurring antioxidant. In this study, a total of 4446 differentially expressed genes (2097 down‐regulated and 2349 up‐regulated) were identified *via* RNA‐Seq in HepG2 cells after exposure to α‐LA for 24 hrs. Moreover, GO and KEGG pathway analyses showed that cancer‐relevant cell membrane proteins were significantly affected. An interaction network analysis predicted that Grb2 might mediate the key target pathways activated by exposure to ɑ‐LA. Verification of the RNA‐Seq and iTRAQ results confirmed that Grb2 mediated the ɑ‐LA‐induced inhibition of cell proliferation *in vitro*. Furthermore, the analysis of human hepatocellular carcinoma specimens obtained from the GEO database showed that the expression of EGFR and Met correlated with that of Grb2. These findings provide a novel mechanism through which ɑ‐LA regulates cell proliferation *via* the down‐regulation of growth factor‐stimulated Grb2 signalling.

## Introduction

Hepatocellular carcinoma (HCC) is the most frequent primary liver cancer and has high morbidity and mortality worldwide. HCCs are strongly related to hepatitis B or C virus infection and chronic liver disease 1. Various treatment options can be considered for HCC 2, and for patients diagnosed at early stages, potentially curative treatments, such as radiofrequency ablation and liver transplantation, are available. Unfortunately, HCC is commonly diagnosed at an intermediate or advanced stage. Conventional non‐surgical cancer treatments rely on the use of cytostatic drugs (such as anthracyclines, alkylating agents and platinum compounds) and/or radiotherapy. The majority of these chemotherapeutics act by inhibiting the synthesis of DNA, RNA and proteins, and the significant therapeutic effects are associated with cells that are in the S and mitotic cell cycle phases. However, cytostatic drugs also target those non‐cancer cells with a high proliferation index, resulting in serious side effects. Cytostatic drugs bind to and chemically modify DNA, leading to chromosomal abnormalities or breaks that can cause mutagenic and carcinogenic effects. Some drugs, such as doxorubicin, have a cumulative dose‐dependent cardiotoxicity, whereas platinum derivatives generally induce nephrotoxicity. Small‐molecule targeted inhibitors and/or tyrosine kinase inhibitors were a breakthrough in the treatment of patients with advanced cancers with target oncogene mutations (*e.g*. epidermal growth factor receptor (EGFR) and Met). However, several patients with oncogene mutations do not show favourable responses to treatment with these targeted inhibitors 3, 4. Thus, the discovery of highly specific features of HCC that can be selectively targeted by novel molecules synthesized in the organism or acquired from dietary sources has attracted great interest in the field of cancer treatment.

Recent studies have shown that antioxidants are promising chemopreventive agents for use in new clinical strategies for treating cancer patients. As an essential cofactor in energy metabolism, alpha lipoic acid (ɑ‐LA) is synthesized in the liver and is a naturally occurring antioxidant 5. ɑ‐LA and its reduced form, dihydrolipoic acid (DHLA), are direct scavengers of free radicals and act indirectly as metal chelators, thereby lessening free‐radical damage. Moreover, ɑ‐LA not only is important to the function of some crucial enzymes that feed carbon into the tricarboxylic acid cycle but also generates dynamic regulatory information about the metabolic status of the mitochondrial matrix, ultimately functioning to control metabolic fluxes 6, 7. A study by German physicians noted reduced levels of ɑ‐LA in the serum of patients with cirrhosis, diabetes mellitus and polyneuropathy as early as the 1960s 8, 9. Recently, ɑ‐LA has been shown to improve insulin sensitivity, vasodilation and polyneuropathy in patients with diabetes mellitus 10. In addition, ɑ‐LA‐related regulatory processes are systematically redesigned in tumour cells, and the affected enzymes commonly become central to cancer metabolism. Some studies have obviously indicated that the effects of ɑ‐LA treatment, such as changes in cell proliferation or apoptosis, are the consequences of changes in tumour cell redox status. Additionally, the products of glycolysis and glutamine‐derived alpha‐ketoglutarate account for the vast majority of carbon entering mitochondrial metabolism in tumours. However, distinct regulatory states respond to the chemical status of pyruvate dehydrogenase, and alpha‐ketoglutarate dehydrogenase enzyme complexes are closely linked to LA 11. Therefore, the uniquely central role of ɑ‐LA in the tumour‐specific regulation of mitochondrial metabolism indicates that targeting lipoate‐responsive control mechanisms might provide a promising new clinical option for anticancer treatments.

The rapid development of transcriptomic and proteomic technologies and the accumulation of vast amounts of model organism genome sequence information have provided an unprecedented opportunity for model organism genomic profiling. In this study, we combined the use of an RNA sequencing (RNA‐Seq) database and isobaric tag for relative and absolute quantitation (iTRAQ)‐based quantitative proteomics data to investigate the dynamic genomic/proteomic response of human hepatoma (HepG2) cells to α‐LA stress. Together with supporting data showing α‐LA‐induced suppression of cell proliferation *in vitro* and the correlation of Met and EGFR expression with growth factor receptor‐bound protein 2 (Grb2) expression *in vivo*, our results demonstrate that α‐LA regulates cell proliferation by altering growth factor‐stimulated Grb2 signalling.

## Materials and methods

### Cell culture and treatment

The human HCC cell line HepG2 was obtained from the American Type Culture Collection (ATCC; Manassas, VA, USA). The cells were cultured in DMEM/F12 complete medium and collected for analysis after treatment with 2.0 mM ɑ‐LA (purity ≥99%, assessed *via* high‐performance liquid chromatography (HPLC), purchased from Sigma‐Aldrich, St. Louis, MO, USA) at 24 hrs.

### RNA isolation and transcriptome analyses

Total RNA was extracted from the cell samples using TRIzol Reagent (Invitrogen Inc., Carlsbad, CA, USA). The resulting samples were treated with DNase I to remove any genomic DNA. RNA samples with A260/A280 ratios between 1.9 and 2.1, RNA 28S:18S ratios greater than 1.0, and RNA integrity numbers (RINs) ≥8.5 were used in the subsequent analyses.

The RNA‐Seq libraries were generated using Illumina TruSeq RNA Sample Preparation Kits following the manufacturer's instructions. Sequencing was conducted on an Illumina HiSeq 2000 platform. The raw reads generated from sequencing were cleaned by removing the adaptor sequences (ATCTCGTATGCCGTC) using an in‐house method 12. We then carried out a stringent low‐quality filtering process. First, bases with a Phred quality score lower than 20 were trimmed from the 3′ end of the sequence until a base with a higher quality score (≥20) was encountered. If the read length was shorter than 50 bp, the read was discarded. Second, reads were further filtered based on the criterion that 50% of the bases in one read must have high‐quality scores (≥10). Third, only paired‐end reads were used for further assembly.

The RNA‐Seq reads were mapped to the human genome using TopHat (version 2.0.9, reference hg19). Cufflinks software (version 2.1.1) was used to identify the differentially expressed genes (DEGs). Transcript abundance was determined as fragments per kilobase of exon per million fragments mapped (FPKM). Samples with zero values across more than 50% of the genes were excluded. Only genes with an adjusted *P*‐value (*q*‐value) <0.05 were considered.

GO and KEGG enrichment analyses of the DEGs were performed using the online tool DAVID (https://david.ncifcrf.gov/). GO terms and KEGG pathways with a Bonferroni corrected *P*‐value of <0.05 were considered significantly enriched function annotations.

### Protein extraction and quantification

Protein extraction from cells was performed as described previously 13. In brief, detached cells were diluted in RIPA buffer containing a protease inhibitor cocktail, incubated on ice for 30 min. and then centrifuged at 18,000g for 30min. at 4°C. The solution was removed from the 2‐ml Zeba™ Thermo Fisher Scientific, Waltham, USA spin columns for desalting, and the resulting desalted protein eluate was collected and quantified using BCA assay (Thermo Fisher Scientific). The protein from each treatment group was aliquoted into 100 μg samples in low‐bind microcentrifuge tubes and dried in a vacuum. The following solutions were then sequentially added: 40 μl of 1 M TEAB buffer, 2 μl of 2% SDS (pH 8.5), 4 μl of tris (2‐carboxyethyl) phosphine and 1 ml of methyl‐methanethiosulfonate. The protein samples were vortexed at each step to ensure that they were thoroughly solubilized. The digestion protein samples were then incubated with 10 ml of a 1‐μg/μl trypsin solution at 37°C overnight. The samples were then dried, solubilized and resuspended to ensure complete solubilization.

Samples were incubated at room temperature for 4 hrs and then desalted and dried. The iTRAQ‐labelled peptides were fractionated using an HPLC system with strong cation exchange (SCX) chromatography (Agilent Technology, Santa Clara, CA, USA). Subsequently, samples were loaded onto a C18 column (Waters Corporation, Milford, MA, USA), and the components produced by SCX chromatography were subjected to tandem mass spectrometry (LC‐MS/MS) analysis.

The raw protein data were examined using the Mascot algorithm and subjected to a search against the human International Protein Index database (version 3.45) using ProteinPilot software (Applied Biosystems, SCIEX, Framingham, MA, USA). To reduce false‐positive data, a strict cut‐off value of an unused ProtScore >1.3 with at least one peptide with 95% confidence limits was applied for protein identification. Only proteins with a ratio greater than 4 and a *P*‐value <0.05 were considered differentially expressed proteins (DEPs).

The interaction relationship of the 72 proteins was retrieved from the STRING database (http://string-db.org/cgi/) and was graphed using Cytoscape software (version 3.2.1). The known network of Grb2 was obtained from the BioGRID database (https://thebiogrid.org/), with MINIMUM EVIDENCE = 10. The heatmaps were visualized using R software (version 3.3.2).

### Cholecystokinin (CCK‐8) assay

For the CCK‐8 assay, cells were seeded at a density of 5 × 10^3^ cells per well in 96‐well plates for 24 hrs and subsequently cultured in the presence or absence of different ɑ‐LA concentrations for 24–120 hrs. At the indicated times, the medium was replaced with fresh medium supplemented with CCK‐8, and the cells were further incubated for 2 hrs. The absorbance at 450 nm was tested using a Thermo microplate reader (Waltham, MA, USA). At least six samples were simultaneously assessed in each experiment, and each experiment was performed at least three times.

### Transient transfection

Knock‐down of Grb2 expression was achieved using the Grb2‐specific siRNA siGrb2‐1 purchased from Cell Signaling Technology (Danvers, MA, USA). siGrb2‐2 (5′‐CAU GUU UCC CCG CAA UUA UTT‐3′) was synthesized at GenePharma (Suzhou, China). For the overexpression studies, the full‐length Grb2 sequence was purchased from Vigene (Rockville, MD, USA) and subcloned into the pENTR expression vectors.

Transient transfections were performed using Lipofectamine 2000 (Invitrogen). Briefly, the cells in 6‐well plates were transfected with siGrb2 or pENTER‐Grb2. Lipofectamine 2000 was incubated with DNA or RNA in serum‐free medium for 20 min. before being added to cells, and the resulting cell suspension was subsequently incubated for an additional 6 hrs. After treatment at the indicated time‐points, the cells were collected for proliferation or Western blot analyses.

### Western blotting

The protein lysates were separated on 10% SDS‐PAGE gels and transferred to PVDF membranes. The membranes were probed overnight at 4°C using primary antibodies against human Grb2 (Abcam, 1:5000 Abcam, Cambridge, MA, USA), EGFR/phospho‐EGFR (Tyr1068), Met/phospho‐Met (Tyr1234/1235), protein kinase B (AKT)/phospho‐AKT (Ser473), cyclin‐dependent kinase (CDK) 2/4/6, cyclin D3/E1 (Cell Signaling Technology, 1:1000 Beverly, MA, USA) or β‐actin (Cell Signaling Technology, 1:2000) and then incubated with an HRP‐linked secondary antibody (Cell Signaling Technology, 1:1000) for 1 hrs. The signal was detected using an ECL Western blotting detection kit (Thermo), and the results were recorded using a GeneGnome XRQ fluorescence chemiluminescence imager (Syngene).

### Sample data from gene expression omnibus (GEO)

The expression of genes in HCC tissue samples was analysed using microarray data sets available in the NCBI database (GEO accession number GSE20140) and uploaded by the Icahn School of Medicine at Mount Sinai. Gene expression data for tumours and adjacent non‐tumour cirrhotic formalin‐fixed, paraffin‐embedded tissues were conducted using the 6k Transcriptionally Informative Gene Panel for DASL (Illumina, GEO‐GPL5474) (Table S1) 14, 15.

Kaplan–Meier survival estimates (overall survival) were based on survival time (years) and survival status (death or survival) and calculated using the Survival package of R software.

### Statistical analysis

Statistical analyses were performed on data collected from at least three independent experiments. The data are presented as the means ± standard deviations and were analysed using GraphPad Prism 5 software. Comparisons of >2 groups were performed with anova and Tukey's test. Comparisons of two groups were performed with unpaired Student's *t‐*tests. A *P*‐value <0.05 was considered statistically significant.

## Results

### Quantitative transcript profiling

**Figure 1:**
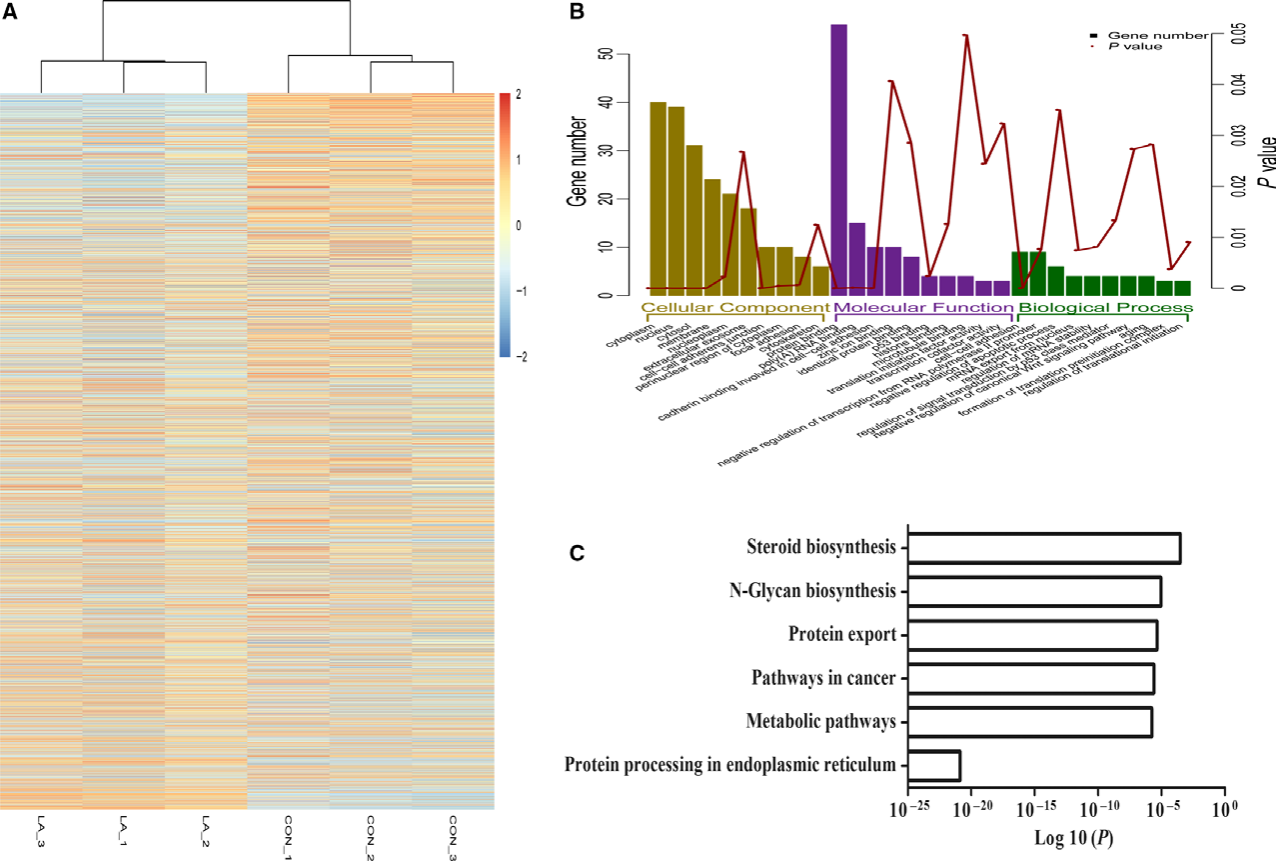
Transcriptome profiling in response to 2 mM α‐LA treatment. (**A)** Heatmap revealing the differentially expressed genes in three replicates of the 24‐hr control‐ and α‐LA‐treated HepG2 cells. Each row represents the relative expression levels of a single gene across all samples. The red blocks represent high expression relative to the control cells, and blue blocks represent low relative expression. (**B** and **C)** GO and KEGG enrichment analysis of the DEGs in HepG2 cells after α‐LA treatment. This analysis was performed using the online tool DAVID (https://david.ncifcrf.gov/).

To study the effect of exposure to α‐LA on the transcriptional profile of HepG2 cells, we conducted RNA‐Seq experiments with triplicate biological replicates extracted from α‐LA‐treated HepG2 cells and control‐treated cells. Approximately 20 million reads per sample were acquired on the Illumina HiSeq™ 2000 platform and aligned to the hg19 reference genome, and more than 26,000 transcripts were detected. DEGs with at least a twofold change in expression and a *P*‐value <0.05 in response to α‐LA treatment were selected. A total of 4446 DEGs were finally identified, including 2097 up‐ and 2349 down‐regulated genes (Fig. 1A, Table S2). We categorized the DEGs according to biological processes, molecular functions and cellular components using GO databases (Fig. 1B). Concerning the cellular components, we determined that 76.73% (2889/3765) of the DEGs were part of the membrane‐bound organelle cellular component. The KEGG analysis revealed that α‐LA obviously affected steroid biosynthesis (*P* = 0.0086), N‐glycan biosynthesis (*P* = 0.0003), protein export (*P* =0.0002), metabolic pathways (*P* = 0.0001), cancer‐relevant pathways (*P* = 0.0001) and protein processing in the endoplasmic reticulum (*P* = 0.0000) (Fig. 1C).

### α‐LA treatment alters expression of proteins in multiple cancer‐relevant pathways

**Figure 2:**
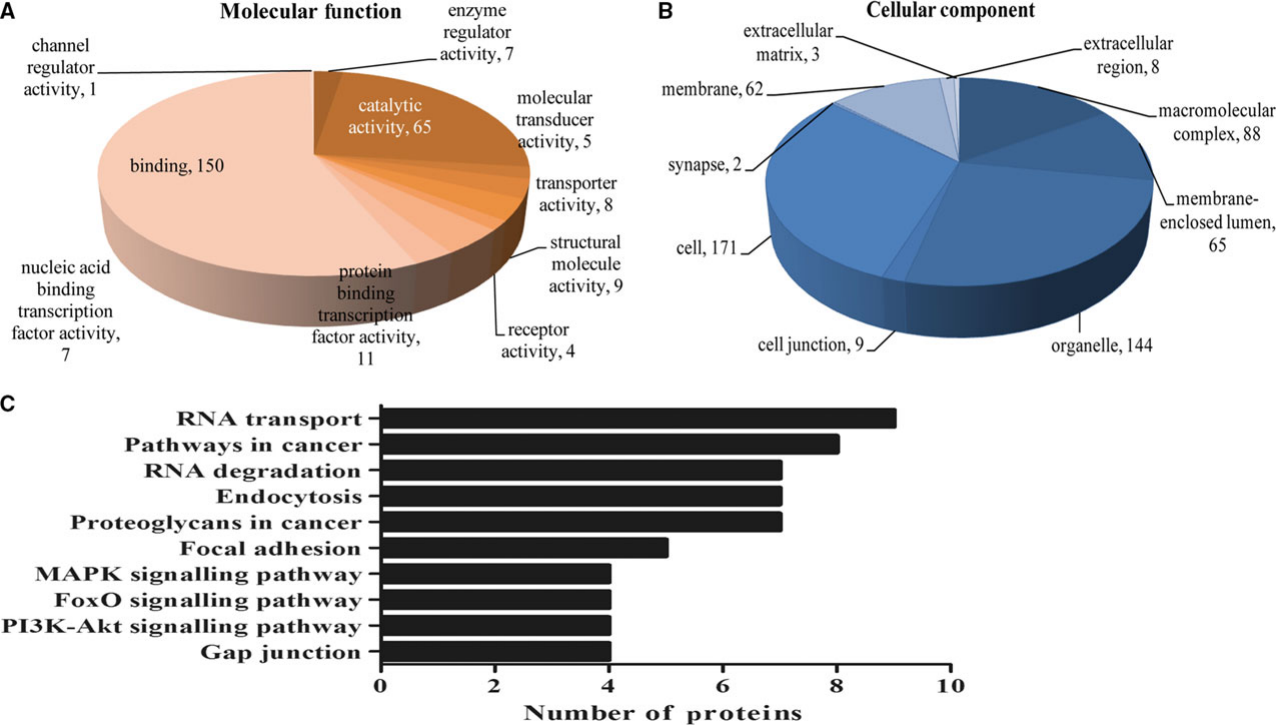
Functional classification of α‐LA‐responsive proteins in HepG2 cells. The proteins were classified into categories for (**A**) molecular function and (**B**) cellular component. (**C**) KEGG pathway analysis of DEP enrichment in HepG2 cells after α‐LA treatment.

The transcriptome data indicated that α‐LA exerts its anticancer function through the regulation of cell membrane structure and function. However, RNAs and proteins are co‐ordinated to regulate cellular process and maintain the homoeostasis of cells. Therefore, a proteome profile analysis was conducted using the iTRAQ‐based quantitative technique to identify information associated with cancer‐related cellular functions. Here, more than 1000 proteins were quantified by LC‐MS/MS. A total of 182 DEPs were identified (Table S3). Binding proteins (95/182) and cell membrane‐related proteins (93/182) significantly altered by α‐LA treatment were functionally annotated to identify their molecular function or cellular component. Using a KEGG analysis, we further explored proteoglycans related to cancer (MAPK14, DDX5, STAT3, EGFR, PXN, GRB2, CBL) and various pathways, including mitogen‐activated protein kinase (MAPK) signalling (MAPK14, DUSP3, EGFR, GRB2), FoxO signalling (MAPK14, STAT3, EGFR, GRB2) and phosphatidylinositol‐4,5‐bisphosphate 3‐kinase (PI3K)‐Akt signalling (CDK4, EGFR, GRB2, LAMA5). Most of the components of these signalling pathways were not only significantly altered by α‐LA treatment but also correlated with tumorigenesis (Fig. 2).

### A protein–protein interaction network reveals new participants in α‐LA‐treated HepG2 cells

**Figure 3:**
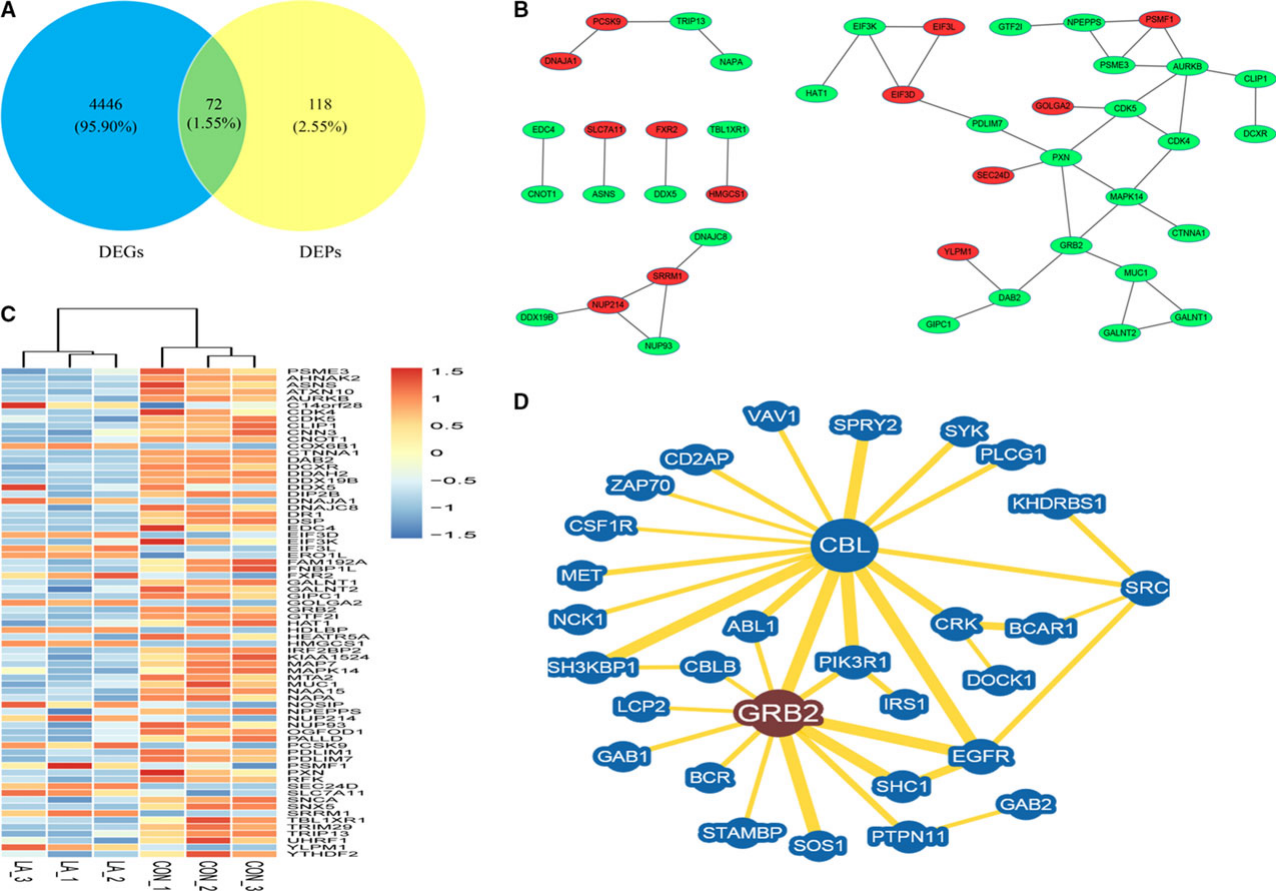
Protein–protein interaction network of DEPs. (**A**) Venn diagram showing the overlapping DEGs (blue) and DEPs (yellow). (**B**) Heatmaps showing the expression variation at the mRNA level of the 72 DEGs/DEPs. (**C**) Protein–protein interactions among these 72 DEGs/DEPs according to the STRING database (http://string-db.org/). Line segments indicate protein–protein interactions, red indicates up‐regulated DEGs/DEPs, and green indicates down‐regulated DEGs/DEPs. (**D**) Grb2‐centred protein–protein interaction network. The network was constructed with the BioGRID database (https://thebiogrid.org/) and used to predict the protein network related to Grb2.

To more comprehensively understand how α‐LA mediates the components of the complex web of interactions among the aforementioned signalling pathways, we first matched all DEGs with quantifiable proteins using the following steps: (*i*) the transcripts representing the proteins included in the proteomics analysis were identified, (*ii*) those transcripts that show differentiation between the α‐LA‐treated HepG2 cells and control‐treated cells at a *P*‐value <0.05 were then identified, and (*iii*) among these significantly affected transcripts, the represented proteins that showed 1.5‐fold up‐ or down‐regulated were identified. These steps resulted in the identification of a total of 72 DEGs/DEPs (Fig. 3A and B, Table 1). We then created an interaction network using our novel proteome/transcriptome‐matched data. The interaction network contained 43 (representing the DEGs/DEPs) nodes and 44 edges (Fig. 3C). Among the nodes, Grb2 had the second‐most degrees of interaction. Interestingly, we observed that Grb2 was significantly down‐regulated at the transcriptional and post‐translational levels and that the expression of the oncogenes with which Grb2 interacts, such as EGFR, Erbb2, Met, SRC and Sos, was changed at the transcriptional and/or post‐translational level by α‐LA treatment (Fig. 3D, Table 1).

**Table 1:** List of overlapping DEGs/DEPs

Gene symbol	Description	*P*‐value of mRNA	E value of protein
MUC1	Mucin‐1	5.000E‐05	0.000E+00
GALNT2	Polypeptide N‐acetylgalactosaminyltransferase 2	7.500E‐04	0.000E+00
IRF2BP2	Interferon regulatory factor 2‐binding protein 2	5.000E‐05	0.000E+00
SRRM1	Serine/arginine repetitive matrix protein 1	7.000E‐04	2.605E‐135
DNAJC8	DnaJ homolog subfamily C member 8	5.000E‐05	0.000E+00
YTHDF2	YTH domain‐containing family protein 2	1.950E‐03	0.000E+00
PCSK9	Proprotein convertase subtilisin/kexin type 9	2.800E‐03	0.000E+00
DR1	Protein Dr1	7.000E‐03	6.544E‐126
FNBP1L	Formin‐binding protein 1‐like	5.000E‐05	0.000E+00
CNN3	Calponin‐3	3.550E‐03	0.000E+00
HAT1	Histone acetyltransferase type B catalytic subunit	2.000E‐04	0.000E+00
HDLBP	Vigilin	5.000E‐05	0.000E+00
KIAA1524	Protein CIP2A	8.050E‐03	0.000E+00
TBL1XR1	F‐box‐like/WD repeat‐containing protein TBL1XR1	5.000E‐05	0.000E+00
SEC24D	Protein transport protein Sec24D	5.000E‐05	0.000E+00
SLC7A11	Cystine/glutamate transporter	9.600E‐03	0.000E+00
NAA15	N‐alpha‐acetyltransferase 15, NatA auxiliary subunit	8.650E‐03	0.000E+00
PALLD	Palladin	4.400E‐03	0.000E+00
SNCA	Alpha‐synuclein	5.000E‐05	1.808E‐93
CTNNA1	Catenin alpha‐1	5.000E‐05	0.000E+00
PDLIM7	PDZ and LIM domain protein 7	4.000E‐04	0.000E+00
DAB2	Disabled homolog 2	5.000E‐05	0.000E+00
HMGCS1	Hydroxymethylglutaryl‐CoA synthase, cytoplasmic	5.000E‐05	0.000E+00
TRIP13	Pachytene checkpoint protein 2 homolog	5.000E‐04	0.000E+00
MAP7	Ensconsin	5.000E‐05	0.000E+00
DDAH2	N(G),N(G)‐dimethylarginine dimethylaminohydrolase 2	5.000E‐05	0.000E+00
MAPK14	Mitogen‐activated protein kinase 14	4.200E‐03	0.000E+00
DSP	Desmoplakin	1.000E‐04	0.000E+00
CDK5	Cyclin‐dependent‐like kinase 5	2.000E‐04	0.000E+00
GTF2I	General transcription factor II‐I	5.000E‐05	0.000E+00
ASNS	Asparagine synthetase [glutamine‐hydrolysing]	5.000E‐05	0.000E+00
GOLGA2	Golgin subfamily A member 2	2.500E‐04	0.000E+00
NUP214	Nuclear pore complex protein Nup214	8.000E‐04	0.000E+00
DNAJA1	DnaJ homolog subfamily A member 1	5.000E‐05	0.000E+00
RFK	Riboflavin kinase	5.000E‐05	2.160E‐114
PDLIM1	PDZ and LIM domain protein 1	5.000E‐05	0.000E+00
TRIM29	Tripartite motif‐containing protein 29	5.000E‐05	0.000E+00
MTA2	Metastasis‐associated protein MTA2	5.000E‐05	0.000E+00
PXN	Paxillin	1.155E‐02	0.000E+00
CLIP1	CAP‐Gly domain‐containing linker protein 1	1.300E‐03	0.000E+00
DIP2B	Disco‐interacting protein 2 homolog B	5.000E‐05	0.000E+00
CDK4	Cyclin‐dependent kinase 4	1.160E‐02	0.000E+00
AHNAK2	Protein AHNAK2	5.000E‐05	0.000E+00
HEATR5A	HEAT repeat‐containing protein 5A	5.000E‐05	0.000E+00
C14orf28	Uncharacterized protein C14orf28	6.550E‐03	0.000E+00
ERO1L	ERO1‐like protein alpha	8.000E‐04	0.000E+00
YLPM1	YLP motif‐containing protein 1	5.500E‐04	0.000E+00
OGFOD1	Prolyl 3‐hydroxylase OGFOD1	6.000E‐04	0.000E+00
NUP93	Nuclear pore complex protein Nup93	2.000E‐04	0.000E+00
FAM192A	Protein FAM192A	5.550E‐03	0.000E+00
CNOT1	CCR4‐NOT transcription complex subunit 1	1.000E‐04	0.000E+00
EDC4	Enhancer of mRNA‐decapping protein 4	1.600E‐03	0.000E+00
DDX19B	ATP‐dependent RNA helicase DDX19B	5.000E‐05	0.000E+00
PSME3	Proteasome activator complex subunit 3	3.500E‐04	3.880E‐178
NPEPPS	Puromycin‐sensitive aminopeptidase	7.150E‐03	0.000E+00
DDX5	Probable ATP‐dependent RNA helicase DDX5	1.700E‐03	0.000E+00
GRB2	Growth factor receptor‐bound protein 2	7.950E‐03	6.238E‐164
FXR2	Fragile X mental retardation syndrome‐related protein 2	5.000E‐05	0.000E+00
DCXR	L‐xylulose reductase	5.000E‐05	1.735E‐179
AURKB	Aurora kinase B	1.000E‐03	0.000E+00
GALNT1	Polypeptide N‐acetylgalactosaminyltransferase 1	5.000E‐05	0.000E+00
GIPC1	PDZ domain‐containing protein GIPC1	5.000E‐05	0.000E+00
COX6B1	Cytochrome c oxidase subunit 6B1	1.000E‐04	7.228E‐62
EIF3K	Eukaryotic translation initiation factor 3 subunit K	9.650E‐03	8.420E‐165
NAPA	Alpha‐soluble NSF attachment protein	4.550E‐03	0.000E+00
UHRF1	E3 ubiquitin‐protein ligase UHRF1	3.900E‐03	0.000E+00
NOSIP	Nitric oxide synthase‐interacting protein	5.000E‐05	0.000E+00
PSMF1	Proteasome inhibitor PI31 subunit	3.350E‐03	0.000E+00
SNX5	Sorting nexin‐5	1.450E‐03	0.000E+00
EIF3D	Eukaryotic translation initiation factor 3 subunit D	5.000E‐05	0.000E+00
EIF3L	Eukaryotic translation initiation factor 3 subunit L	5.000E‐05	0.000E+00
ATXN10	Ataxin‐10	8.500E‐04	0.000E+00

### Potential mechanism of Grb2 in α‐LA‐reduced cell proliferation

**Figure 4:**
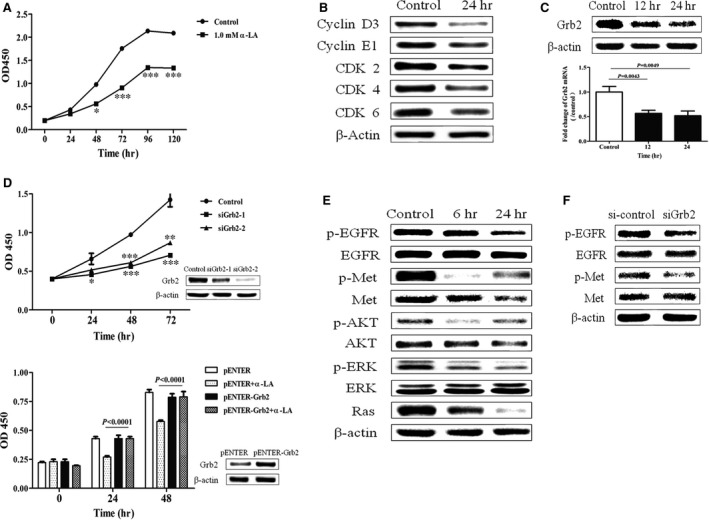
Grb2 mediates the ɑ‐LA‐induced reduction in cell proliferation. (**A**) HepG2 cell proliferation was measured through a CCK‐8 assay at the indicated times. (**B and C**) Cell cycle regulatory protein expression in HepG2 cells after treatment with 2.0 mM ɑ‐LA for 24 hrs was assessed *via* Western blotting. (**D**) Grb2 levels were measured *via* real‐time PCR and Western blotting in HepG2 cells after treatment with 1.0 mM ɑ‐LA for 12 and 24 hrs (upper panel). HepG2 cells were transfected with siRNA against Grb2, and 24 hrs after transfection, the cells were seeded into 96‐well plates for CCK‐8 assays at the indicated times (middle panel). After transfection with Grb2 overexpression plasmids, cell proliferation was measured at the indicated times through a CCK‐8 assay (lower panel). (**E**) A Western blotting assay was performed to assess the levels of phosphorylated EGFR, Met, ERK and Ras in HepG2 cells after treatment with 2.0 mM ɑ‐LA for 6 and 24 hrs. (**F**) HepG2 cells were transfected with 50 nM scramble siRNA or Grb2 siRNA (siGrb2) for 48 hrs and then treated with ɑ‐LA for 24 hrs, and the levels of phosphorylated EGFR and Met were analysed *via* Western blotting.

To confirm the above‐described findings, the effect of ɑ‐LA on the proliferation of HepG2 cells was examined. As shown in Figure 4A, 24‐hr treatment with ɑ‐LA significantly repressed HepG2 cell proliferation, as measured by the CCK‐8 assay. In addition, the Western blot assay confirmed that cyclin D1 and cyclin E, which are essential for the control of the cell cycle at the G1/S transition, were down‐regulated by α‐LA treatment (Fig. 4B). Importantly, we found that 12‐ and 24‐hr ɑ‐LA treatments significantly inhibited Grb2 expression in HepG2 cells at both the mRNA and protein levels (Fig. 4C). Knock‐down of Grb2 expression remarkably attenuated cell proliferation; in contrast, Grb2 overexpression completely reversed the cell proliferation blockade induced by ɑ‐LA treatment in HepG2 cells (Fig. 4D). Similar results were observed in the A549, NCI‐H975 (data not shown) and HCT116 cell lines (Fig. S1). Furthermore, ɑ‐LA clearly blocked EGFR, Met, AKT and ERK 1/2 phosphorylation, and the levels of Ras were notably decreased with 24‐hr treatment (Fig. 4E). As shown in Figure 4F, the treatment of HepG2 cells with specific siRNAs against Grb2 expression significantly attenuated the levels of phospho‐EGFR and phospho‐Met. These results also indirectly suggest that Grb2 mediates the ɑ‐LA‐induced modulation of cell proliferation through targeting EGFR/Met/Ras‐MAPK/AKT signalling.

### Grb2 overexpression is closely associated with a poor prognosis in HCC

**Figure 5:**
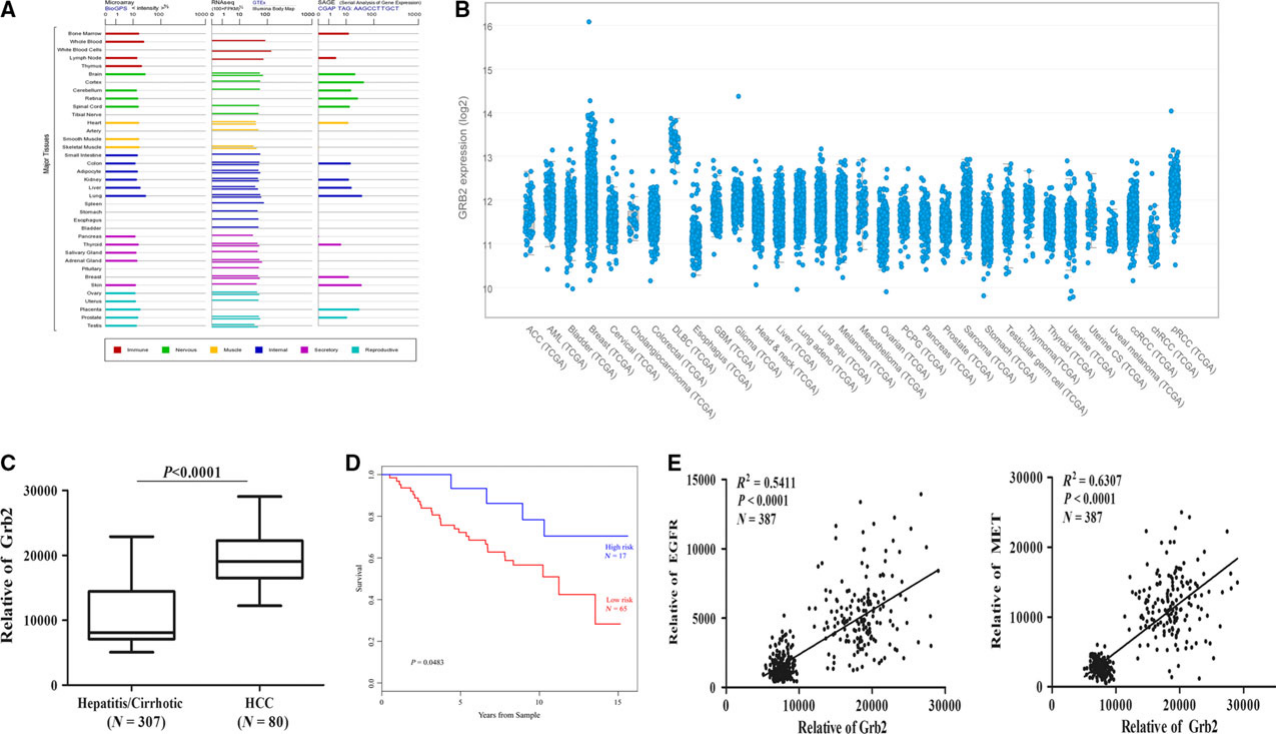
Correlations of EGFR, Met and Grb2 in human HCC specimens. (**A**) Grb2 expression in normal tissue downloaded from the GeneCards website. (**B**) Grb2 expression in various cancers queried from the cBioPortal database. (**C**) The expression of Grb2 in HCC and adjacent tissue (NCBI GEO accession number 20140) was analysed. (**D**) Kaplan–Meier curves showing survival times according to the Grb2 signature in HCC patients. The patients with expression above the median are shown in red, and the patients with expression below the median are shown in blue. (**E**) A Pearson's correlation analysis of Met/Grb2 and EGFR/Grb2 expression from microarrays of human hepatic malignant lesions tissue (NCBI GEO accession number 20140), which provided data from 287 specimens, including a training cohort (80 tumour and 82 non‐tumour liver samples) and a validation cohort (225 non‐tumour liver tissue samples surgically resected from patients with HCC), was performed.

Because Grb2 expression was found to be regulated by α‐LA, it is important to evaluate the clinical significance of the abnormal expression of Grb2 in HCC. Grb2 was shown to be highly and ubiquitously expressed in major tissues (Fig. 5A) and various cancers (Fig. 5B), as shown by data obtained from the GeneCards and cBioPortal database, respectively. In addition, the expression of Grb2 in HCC tissue samples was analysed using the NCBI database (GEO accession numbers GSE20140‐GPL5474) 13, 14 (Table S3). For this analysis, we compared the Grb2 levels in the HCC specimens with those in the adjacent non‐tumour data sets and found that Grb2 was more highly expressed in the HCC tissue than in the non‐tumour specimens (Fig. 5C). Kaplan–Meier analysis revealed that the high levels of Grb2 expression in the hepatic malignant lesions tissue were associated with lower survival rates (Fig. 5D). Furthermore, we found that the expression levels of EGFR and Met were positively correlated with the expression of Grb2 in precancerous/cancerous tissues (Fig. 5E). These results emphasize the importance of Grb2 expression in HCC progression.

## Discussion

Cancer is a complex multi‐step process arising from a combination of genetic and epigenetic alterations, somatic mutations, genomic instability and environmental factors. Many non‐conventional anticancer drugs have recently been introduced due to their substantial inhibitory activity against the proliferation of many type of cancers *in vitro* and against tumour carcinogenesis *in vivo* 16.

ɑ‐LA is a biological antioxidant currently used to treat clinical diseases such as diabetes, Alzheimer's disease and peripheral neuropathy. Recent *in vitro* experiments have revealed that ɑ‐LA exhibits inhibitory effects against multiple types of cancer cells through the suppression of cell proliferation or the induction of apoptosis but does not affect normal cell viability at the same concentration 17. This evidence suggests that the mechanism through which ɑ‐LA inhibits the growth of cancer cells is different from that of normal cells in the same tissue. However, the specific mechanisms involved in the cellular response to ɑ‐LA have not been fully addressed. Our study constitutes the first investigation of the expression patterns of transcripts combined with the proteomics profile to search for a key mediator in HCC cells when exposed to α‐LA stress. We conducted bioinformatic analyses of the integrated data to examine the α‐LA‐mediated alterations, with a focus on cell membrane and/or binding proteins that influence the basic cell communication systems and multiple signalling transduction pathways in human hepatic cells.

**Figure 6:**
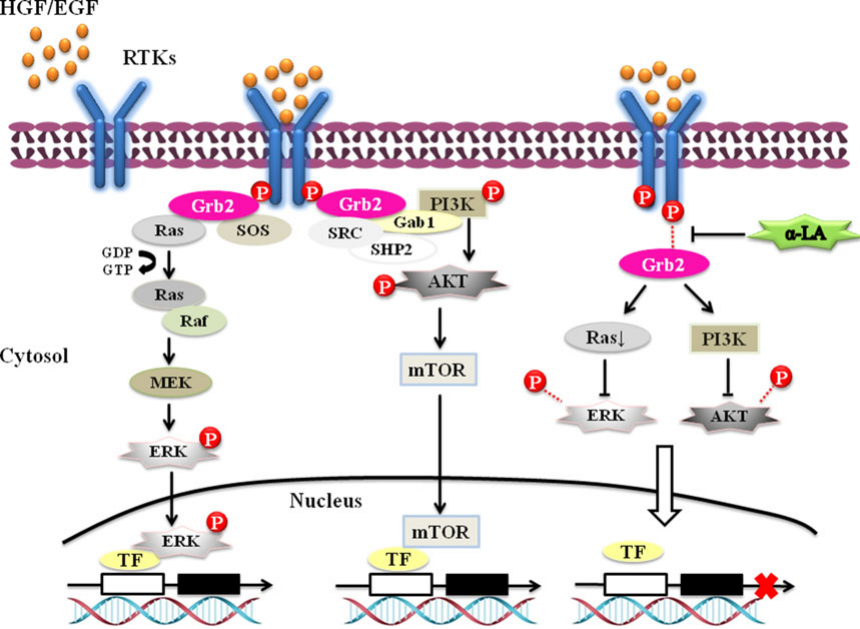
Schematic diagram illustrating how ɑ‐LA, *via* regulation of Grb2 expression, modulates different signalling pathways that participate in proliferation‐related stress responses. Growth factors (EGF/HGF) in the extracellular matrix bind to RTKs on the plasma membrane and subsequently phosphorylate the docking site and recruit effector molecules [Grb2, Grb2‐associated‐binding protein 1 (Gab 1), SRC homology 2 domain‐containing phosphatase 2 (SHP2), Son of Sevenless (Sos) and sarcoma non‐receptor tyrosine kinase (SRC)]. ɑ‐LA, by down‐regulating Grb2 expression, impairs the phosphorylation of the docking site of effector molecules and then subsequently attenuates the downstream ERK/MAPK pathway and the PI3K‐AKT pathway, leading to decreased ERK1/2 and/or mTOR translocation across the nuclear membrane and/or inhibition of the activation of transcription factors (TFs) and gene expression. The signals affect gene expression and repress cell proliferation and survival, resulting in the arrest of cancer growth and progression.

Receptor tyrosine kinases (RTKs) are known to provide basic communication systems between the extracellular milieu and intracellular signalling pathways. Signalling pathways mediated by RTKs also play important roles in the development and progression of human cancers. Interestingly, the RNA‐Seq or proteomics data showed no obvious alterations in EGFR and Met expression at the mRNA and proteins levels after α‐LA treatment. However, we tested whether activation of EGFR and Met was down‐regulated in HepG2 cells by 6‐ and 24‐hr α‐LA treatment. These results provided insight into whether α‐LA inhibits hepatic cell growth through the regulation of EGFR or Met activation and the targets that mediate this process. Certain proteins act as signal transducers that are recruited to sites of tyrosine kinase activation by interactions with the plasma membrane or protein–protein interactions. According to the results described above, the protein–protein interaction networks (Fig. 3C and D) uncovered Grb2 as a key player among the groups of DEGs/DEPs. Grb2 possesses a membrane‐targeting region at the N terminus and multiple tyrosine phosphorylation sites that function as binding sites for SRC homology domains of a variety of downstream effectors. Grb2 uses the N‐terminal SRC homology 3 (SH3) domain to bind the polyproline sequence in Sos and Grb2‐associated‐binding protein 1 (Gab 1), increasing the possibility of the translocation of Sos or Gab 1 to the membrane to activate the Ras‐MAPK and PI3K‐Akt pathways in a ligand‐independent manner (Fig. 6) 18. Grb2‐mediated signalling contributes to a loss of cell cycle control and enhances cell proliferation, motility and invasion 19, and elevated Grb2 expression has been correlated with a poor prognosis and disease progression 20, 21. A study conducted showed that Grb2 inhibition significantly reduces fat accumulation, improves glucose metabolism and ameliorates oxidative stress 22. Using the Gene Expression Omnibus (GEO) database, we found that Grb2 expression was not only closely associated with HCC progression but also positively correlated with EGFR and Met expression in HCC patients (Fig. 5C–E).

The Grb2‐mediated regulation of RTK basal phosphorylation offers potential for the design of a novel class of modulators of related signalling. EGFR is one of the most extensively studied RTKs due to its role in the development of several solid tumours. Inappropriate expression of Met, as the RTK oncogene, mediates signalling dysregulation or hyperactivation and has been reported in human cancers and linked to poor prognoses. Hence, dissecting the EGFR 23 or Met 24 pathways activated by ligand binding is relevant to cancer therapeutics. Some antagonists have been found to bind to the Grb2 SH2 domain to block hepatocyte growth factor (HGF)/EGF‐stimulated Grb2 signalling 25, 26. A particularly useful agent should cross the cell membrane and abrogate the signalling axis and its activation, thereby leading to cell apoptosis or even cellular senescence. α‐LA can cross the cell membrane to exert its effects in aqueous or lipophilic environments. Moreover, α‐LA possesses an eight‐carbon disulphide chemical backbone structure, which is substantially different from that of typical tyrosine kinase inhibitors. Nonetheless, whether the α‐LA‐induced repression of EGFR or Met signalling of HCC cells occurs through attenuation of Grb2‐associated‐binding events to block growth factor‐stimulated Grb2 signalling or an immediate suppression of the activation of EGFR or Met signalling, which limits the growth and proliferation of cells, needs to be explored further.

In summary, the results of this study provide preliminary evidence of the antitumour effects of α‐LA treatment on HCC cells. RNA‐Seq and iTRAQ‐based quantitative proteomics profiling revealed that ɑ‐LA suppresses HepG2 cell proliferation *via* the down‐regulation of Grb2 expression, thereby blocking growth factor signalling transduction. Further studies are needed to validate the multiple functions of α‐LA and provide evidence to support its application in preventive treatments and therapies for human HCC.

## Conflict of interest

The authors declare that they have no conflict of interests concerning the contents of this article.

## Supporting information


**Figure S1.** Grb2 mediates the ɑ‐LA‐induced reduction in HCT116 cell proliferation.

**Table S1.** Clinical data from the Gene Expression Omnibus (GEO).
**Table S2.** List of differentially expressed genes (DEGs).
**Table S3.** List of differentially expressed proteins (DEPs).
