# Supplementary figures and images for: Integrated transcriptomic and proteomic analyses reveal ɑ‐lipoic acid‐regulated cell proliferation via Grb2‐mediated signalling in hepatic cancer cells
Source: J Cell Mol Med. 2018 Mar 25;22(6):2981–92. doi: 10.1111/jcmm.13447 (PMC5980154; doi:10.1111/jcmm.13447)

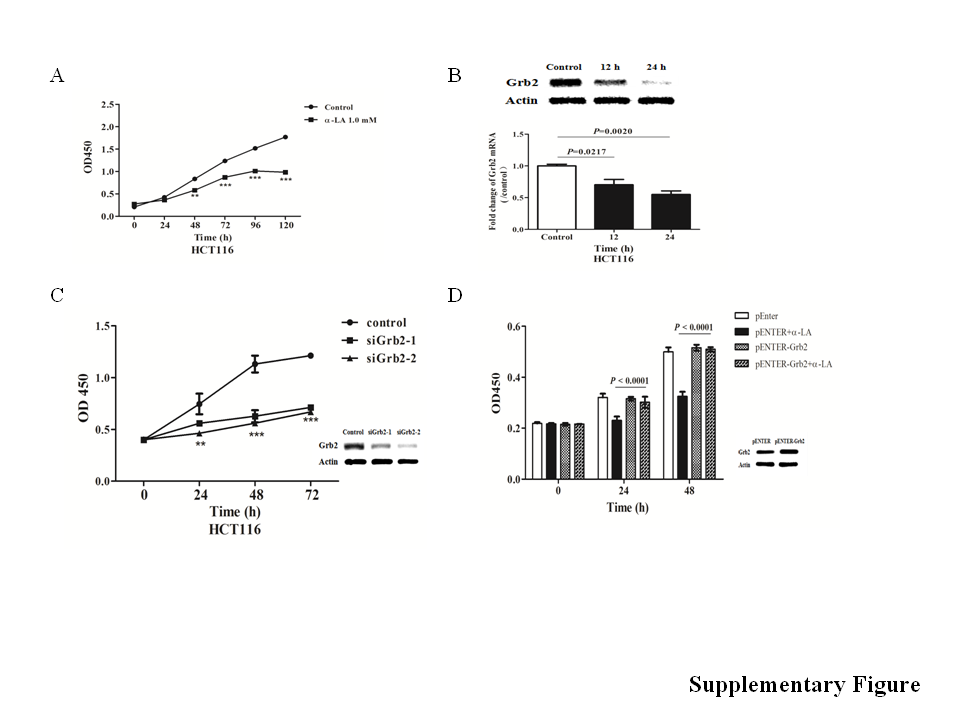

Supplement: Supplementary file 1 — Figure S1. Grb2 mediates the ɑ‐LA‐induced reduction in HCT116 cell proliferation. [file JCMM-22-2981-s001.tif]
